# A novel bioaccessibility-based probabilistic risks assessment of potentially toxic elements (PTEs) in earthworm

**DOI:** 10.3389/fphar.2024.1398394

**Published:** 2024-07-04

**Authors:** Tian-Tian Zuo, Jia-Lin Liu, Hong-Yu Jin, Yan Chang, Feng Wei, Sheng Wei, Shuai Kang, Shuang-Cheng Ma

**Affiliations:** ^1^ National Institutes for Food and Drug Control, Beijing, China; ^2^ Tianjin Center for Disease Control and Prevention, Tianjin, China; ^3^ School of Public Health and Emergency Management, Southern University of Science and Technology, Shenzhen, China; ^4^ Chinese Pharmacopeia Commission, Beijing, China

**Keywords:** earthworm, potentially toxic elements, bioaccessibility, probabilistic risk, Monte Carlo simulation, sensitivity analysis

## Abstract

**Introduction:** Early risk assessment studies usually based on total heavy metal (loid) contents, inevitably leading to an overestimation of the health risks. In addition, inputs are represented as single-point estimates in deterministic models, leading to underestimation or overestimation of the health risks.

**Methods:** To overcome these barriers, a novel probabilistic risk assessment strategy based on the combinational use of bioaccessibility and Monte Carlo simulation was developed to assess heavy metal (loid) associated health risks of earthworms in this study. To obtain a realistic and robust probabilistic risk assessment, heavy metal (loid) exposure duration and frequency were determined using our questionnaire data.

**Results:** As a result, the mean gastrointestinal bioaccessibility was in the order: Cd > As > Cu > Hg. The mean hazard index (HI) values for investigated metal (loid)s were 0.65 and 0.59 for male and female, respectively, demonstrating an acceptable health risk in an average community. However, the 90th percentile of HI values was 1.87 and 1.65 for male and female, respectively. And the total non-cancer risks of heavy metal (loid) exposure exceeded the acceptable threshold for 19.9% and 17.8% of male and female, respectively. In addition, the total cancer risk (TCR) value through co-exposure to As and Cd suggested that the carcinogenic risks may be of concern for average exposure population. Sensitivity analyses revealed that the exposure frequency and bioaccessible As concentration were the dominant contributors to the total risk variance, which provided meaningful implications for environmental management.

**Conclusion:** Altogether, the refined strategy based on bioaccessibility and Monte Carlo simulation is the first of its kind, such effort attempts to scientifically guide the rational clinic use of TCM and the improvement of population-health.

## Graphical Abstract

**Figure:**
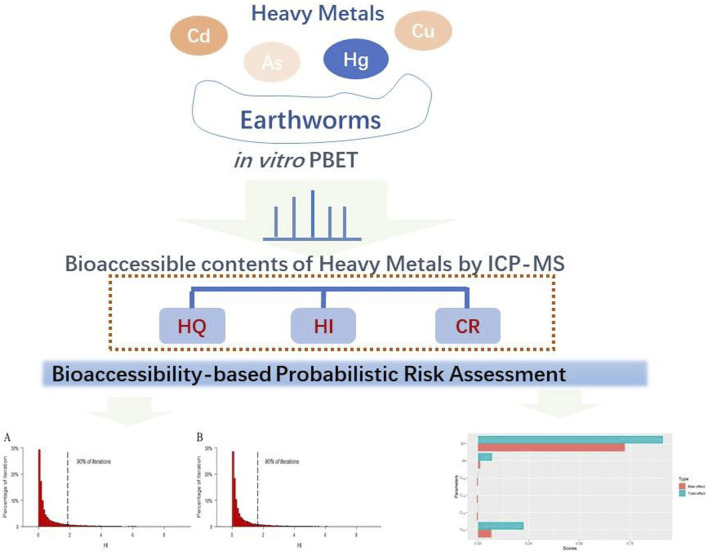
Probabilistic risk assessment strategy of potentially toxic elements in earthworms based on bioaccessibility and MCS technique.

## 1 Introduction

Heavy metal (loid)s including cadmium (Cd), arsenic (As), and mercury (Hg) are potentially toxic elements of global concern due to their long biological half-lives, non-biodegradability, and detrimental effects on humans, even at low levels (Zazouli et al., 2006; Saha et al., 2016; Ghaneian et al., 2017; Tang et al., 2017; Lin et al., 2018). Studies have demonstrated that these potentially toxic elements exert toxic effects by impairing DNA and protein functions, cellular metabolism, and cellular respiration (Sirot et al., 2009). Long-term exposure to excessive levels of potentially toxic elements, including cadmium (Cd), arsenic (As), and mercury (Hg), can induce a series of toxic effects on the central nervous system or internal organs (Li et al., 2014; Ghaleno et al., 2015; Gu et al., 2016). Cd can cause reproductive deficiencies, obstructive pulmonary disease, bone loss, renal dysfunction, and diabetes (Recatalá et al., 2010; Akesson, 2011; He et al., 2013; Chowdhury et al., 2016). As a ubiquitous metalloid, As can induce adverse health effects, including kidney disorders, cardiovascular problems, muscle spasms, hematologic disorders, immune system diseases, and an increased risk of stillbirth (Hughes, 2002; Gilbert-Diamond et al., 2013). In addition, epidemiological studies have demonstrated that As and Cd are carcinogenic and mutagenic without a threshold; it increases the risk of visceral cancer, as well as kidney, bladder, and lung cancer (Kapaj et al., 2006; Dangleben et al., 2013). Hg is highly toxic to the central nervous system and can trigger multiple organ lesions throughout an individual’s lifespan (Bjőrnberg et al., 2003; Schober et al., 2003; Vieira et al., 2011). Although copper (Cu) is an essential trace element for human health, non-carcinogenic physiological toxicity may occur after excessive intake of this metal (Zang et al., 2018; Zhou et al., 2018).

Earthworms are an important animal component used in traditional Chinese medicine (TCM). Given their unique and beneficial activities and curative characteristics, earthworms have long been extensively used for the clinical treatment of a variety of diseases, as described in “Shennong’s Herbal Classic of Materia Medica” in the Donghan dynasty (Qin, 2008; Takahashi et al., 2010). Currently, medicinal earthworms are frequently used in China (Li et al., 2022). The proposed therapeutic benefits of earthworms include anti-thrombotic, anti-arteriosclerosis, anti-arrhythmic, anti-tumor, immune-modulatory anti-hypertension, and diuretic properties (Wang et al., 2019).

Earthworms live in the soil and consume decaying organic matter; thus, they are subject to bioaccumulation of persistent environmental contaminants such as Cd, As, Hg, Cu that are present in both the soil and other organisms. The subsequent ingestion of earthworms contaminated by these potentially toxic elements through the food chain will lead to the accumulation of these contaminants in humans, eventually causing serious toxic symptoms and health risks to human body. Therefore, the continuous evaluation the risk of these potentially toxic elements exposure from the consumption of earthworms is of high significance.

Limited research on potentially toxic elements contamination in earthworms is available. Furthermore, previous studies evaluating environmental contaminants in traditional medicines typically focused on the total contents of potentially toxic elements, based on which deterministic risk assessments were conducted (Sun et al., 2020). However, such determination assumed that amounts of potentially toxic elements released from the matrix to the gastrointestinal tract could be totally absorbed by the human body, which often lead to an overestimation of the exposure contents and potentially health risks to humans. Therefore, better understanding of the bioaccessibility of potentially toxic elements is crucial to assess their health risk against humans. In addition, In the classic deterministic model, inputs are represented as single-point estimates, and the risk outcomes are based on mean values or extreme cases, which may lead to underestimation or overestimation of the risks (Benke and Hamilton, 2008; Yang et al., 2014; Tong et al., 2018). To overcome these barriers, a novel probabilistic risk assessment strategy based on the combinational use of bioaccessibility and Monte Carlo simulation was urgently needed to develop. Monte Carlo simulation describes uncertainty in the model inputs and is the most widely used approaches among the various approaches for applying the probabilistic risk assessment (Farzadkia et al., 2015). In this method, the stochastic behavior of the risk model is explored using the probability distribution of inputs, random numbers, and statistical sampling methods. Moreover, sensitivity analysis allows the user to identify variables with the greatest impact on the risk assessment results, which could be realized by Monte Carlo simulation. Collectively, an innovative health risk assessment of potentially toxic elements in earthworms based on both bioaccessibility and MCS technique was necessary to be explored to provide baseline data for future research in this area.

In order to fill the research gap and scientifically evaluate the safety of earthworms and the associated human health risks, our effort aimed at 1) to investigate the bioaccessible levels of Cd, As, Hg, and Cu in earthworms; 2) to innovate a probabilistic health risk assessment approach for earthworms by considering both the bioaccessibility of potentially toxic elements and uncertainty in crucial exposure factors by the comprehensive use of estimated daily intake (EDI), hazard quotient (HQ), hazard index (HI), and cancer risk (CR) scales; 3) to conduct a sensitivity analysis of exposure parameters duration probabilistic risk assessment; 4) to provide novel insights for remediation strategies in order to improve ecological and public health. Our novel health risk assessment considered both the bioaccessibility of potentially toxic elements and uncertainty in crucial exposure factors through the comprehensive use of estimated daily intake (EDI), hazard quotient (HQ), hazard index (HI), and cancer risk (CR) scales for the first time.

## 2 Material and methods

### 2.1 Sample collection

**FIGURE 1:**
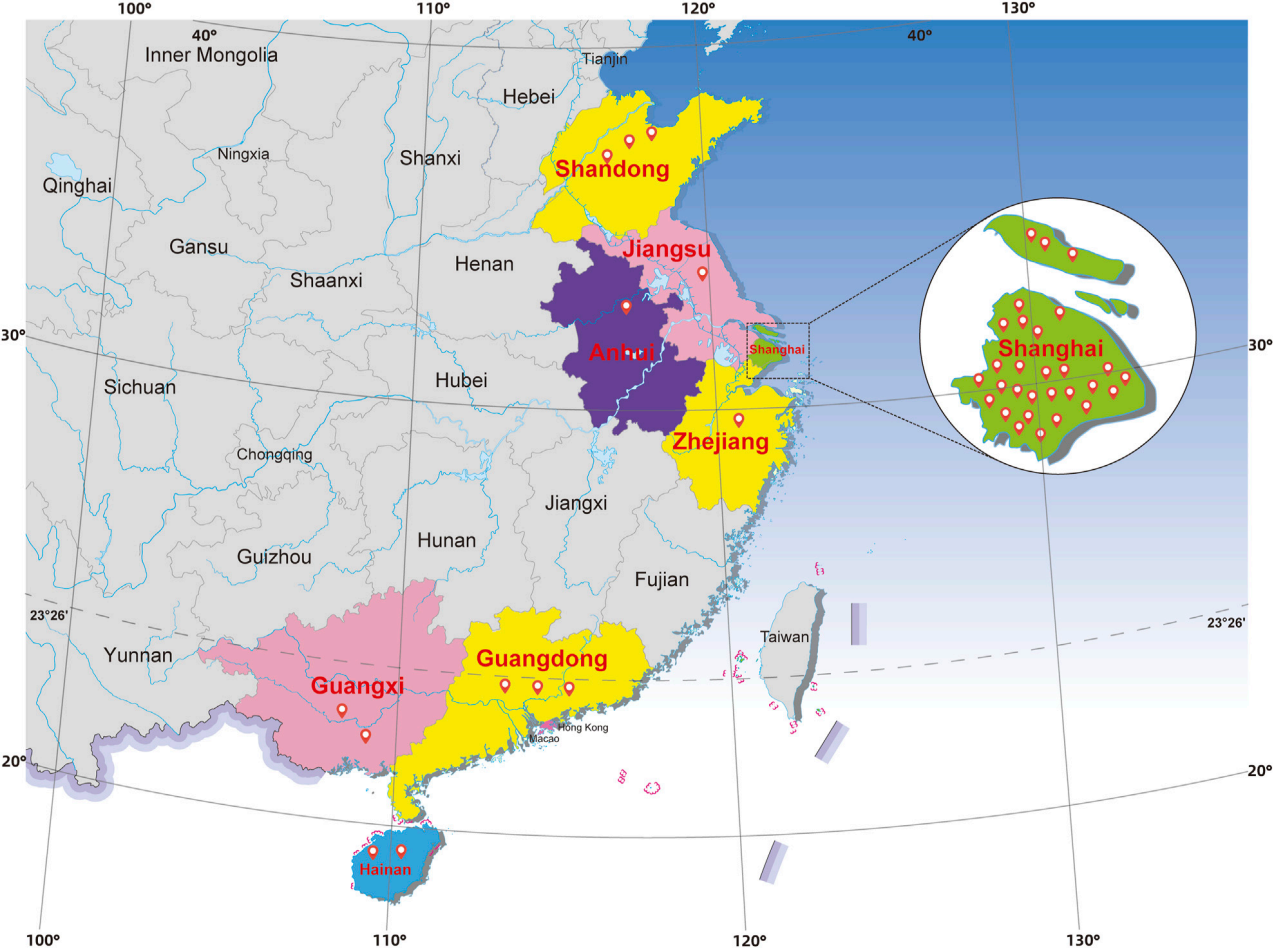
Location of the sampling areas.

A total of 42 batches of dried body of earthworms were collected from retail pharmacies and traditional Chinese medicine (TCM) markets in the provinces of Guangdong, Guangxi, Shandong, Hainan, Zhejiang, Anhui, Jiangsu, and the city of Shanghai (Figure 1). The collection sites reflected different environmental zones in China (110° 12′E, 118° 70′E, and 19° 07′N to 37° 45′N). All earthworm samples were authenticated by Dr. Shuai Kang. Voucher specimens were deposited in the National Institutes for Food and Drug Control (NIFDC), Beijing, China.

### 2.2 Analysis of total concentrations of potentially toxic elements

All the samples were carefully rinsed in water and kept dry in moisture-proof plastic bags at 4°C until analysis. Then earthworm samples were ground into fine homogenous powder, and each sample (0.5 g) was placed in a microwave digestion tube (CEM Corporation, Matthews, NC, United States) and digested with 8.0 mL HNO_3_ (Merck, Munchen, Germany). The microwave digestion program was as follows: heating for 3 min to 120°C and holding for 3 min, heating for 2 min to 150°C and holding for 3 min, and heating for 3 min to 190°C and holding for 15 min. After digestion, the excess acid was removed and the digested solution was diluted to 50.0 mL with deionized water prior to determination. The levels of Cd, As, Hg, and Cu in the samples were measured using an Agilent 7700X inductively coupled plasma mass spectrometry (ICP-MS, Agilent 7700X, Agilent Technologies Co., United States).

### 2.3 Quality assurance and quality control

For quality control of the analytical procedures, chemical blanks, duplicates, and spikes were measured throughout the determination process. All samples were analyzed in duplicate. The mean recovery rate of the samples (*n* = 9) was used to control the accuracy of the method. The mean percentage recoveries of Cd, As, and Hg in the earthworms were 98.0% ± 0.92%, 103.4% ± 0.587%, and 89.0% ± 6.21%, respectively. The certified reference material, citrus leaf, was also measured during the determination process. The contents of Cd, As, Hg, and Cu in the citrus leaf were 1.8 × 10^−4^ mg/kg, 1.2 mg/kg, 0.14 mg/kg, and 6.4 mg/kg, respectively, which exhibited good agreement with the certified levels. In addition, an internal standard was added to the blanks, samples, and calibration standard solutions to compensate for matrix effects and signal drift.

### 2.4 Bioaccessible heavy metal (loid) concentration determination

**FIGURE 2:**
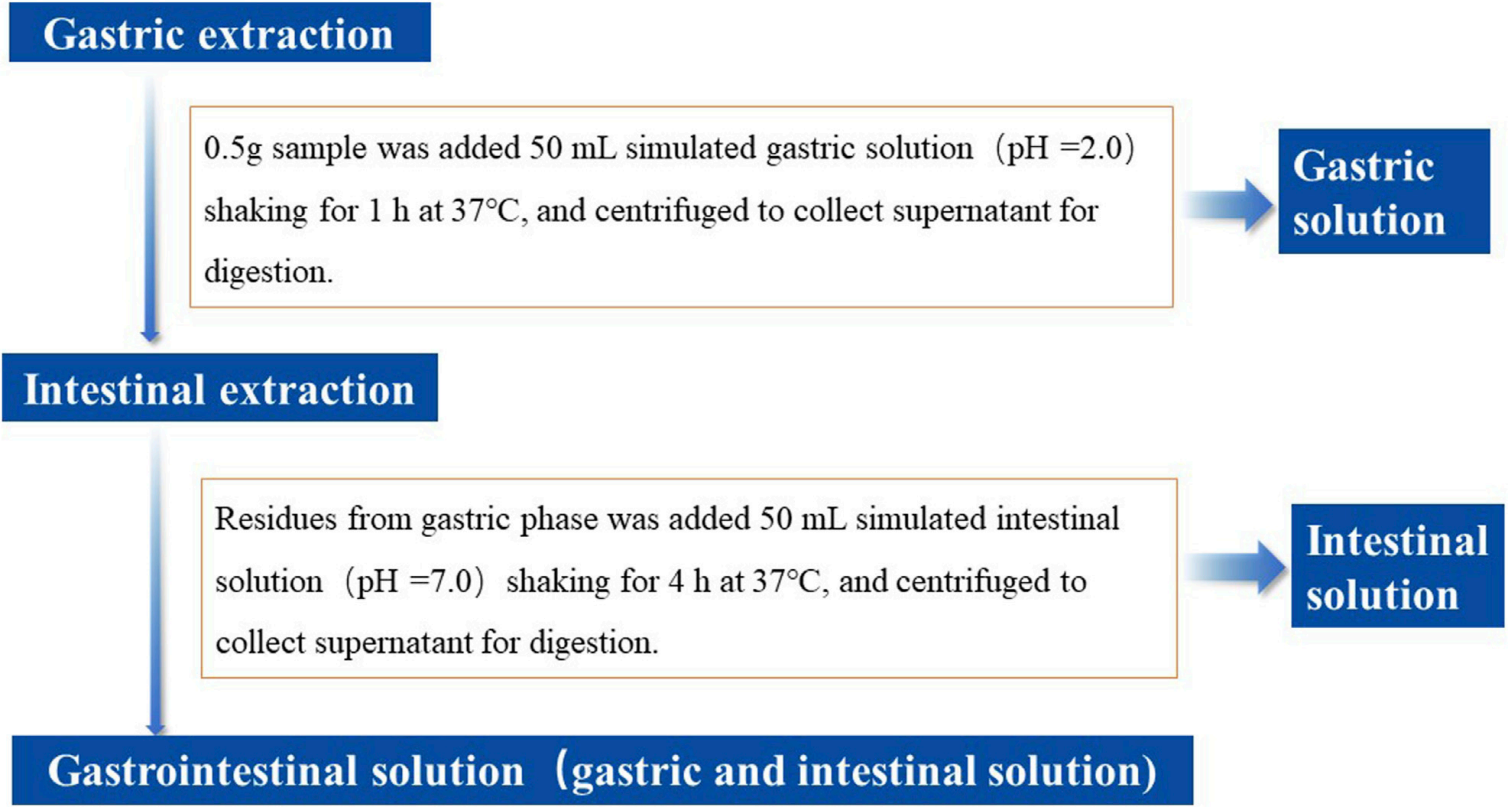
*In vitro* PBET extraction process.

The physiologically based extraction test (PBET) (Ruby et al., 1996), first proposed in 1996, has been recognized as an important method for investigating the bioaccessibility of toxic elements in soil and food. In this study, *in vitro* PBET approach was employed to determine the bioaccessibility of potentially toxic elements in earthworms (Figure 2). The optimized model consisted of a gastric and intestinal extraction phase (Ruby et al., 1996). During the former, the samples (0.5 g) were mixed with 50 mL simulated gastric solution (1.25 g of pepsin, 0.50 g of sodium citrate, 0.50 g of sodium malate, 500 μL of acetic acid, and 420 μL of lactic acid were made up to 1 L by deionized water) and pH was adjusted to 2.0 by HCl. The mixed solutions were then incubated at 37°C, shaken for 1 h, and centrifuged to collect the supernatant. The collected 25 mL supernatant was concentrated to approximately 3 mL at a low temperature using an electro-thermal plate. After cooling, 5 mL of nitric acid was added to the concentrated supernatant for digestion.

During the intestinal extraction stage, the residues from gastric phase were added to 50 mL of simulated intestinal solution (1.75 g of bile salts and 0.5 g of pancreatin made up to 1 L by deionized water) and pH was adjusted to 7.0 by NaHCO_3_ (Zuo et al., 2020a). The solution was incubated and shaken at 37°C for 4 h and centrifuged to collect 25 mL of supernatant. The supernatant was concentrated to approximately 3 mL. After cooling, 5 mL of nitric acid was added to it for digestion via the microwave digestion procedures.

After digestion, deionized water was added to the digested gastric and intestinal solutions till a volume of 50 mL. These solutions were then utilized to analyze potentially toxic elements contents via ICP-MS. The bioaccessibility of potentially toxic elements in earthworms was expressed as the ratio of soluble potentially toxic elements content in a simulated gastrointestinal solution to the total heavy metal (loid) content in the earthworms: using Eq. 1.

$$\text{Bioaccessibility }\left(\%\right)=\frac{\text{bioaccessible}\;\text{heavy}\;\text{metal}\left(\text{loid}\right)\;\text{content}}{\;\text{total}\;\text{heavy}\;\text{metal}\left(\text{loid}\right)\;\text{content}\;}\times 100$$ (1)

where bioaccessible potentially toxic elements content is the sum of soluble heavy metal (loid) (mg/kg) extracted from earthworms in gastric and intestinal phase via the *in vitro* PBET assay and total heavy metal (loid) content is the total heavy metal (loid) (mg/kg) in earthworms.

### 2.5 Exposure assessment

To determine the level of dietary exposure of humans to potentially toxic elements from earthworm consumption, we conducted surveys of 20,917 volunteers (9,420 males and 11,497 females) using face-to-face questionnaires. The EDI was calculated using Eq. 2 (Saha et al., 2016; Kusin et al., 2018).

$$\text{EDI}=\frac{\text{EF}\times \text{Ed}\times \text{IR}\times C\times \text{BA}}{\text{AT}\times W}$$ (2)

where EDI is the estimated daily intake of potentially toxic elements in earthworms (μg/kg/d) and EF is the exposure frequency obtained from the questionnaires. Ed is the exposure duration for traditional animal medicines, which was 20 years, according to the questionnaires; IR is the daily intake rate of earthworms, which is 5–10 g based on the Chinese Pharmacopoeia (The Pharmacopoeia Commission of the People’s Republic of China, 2020), following the uniform distribution; C is the concentration of Cd, As, Hg, or Cu, which was detected in samples by ICP-MS (mg/kg); BA represents the bioaccessibility of potentially toxic elements; AT is the average exposure time to earthworms, which was equal to 365 days/year × 70 years; and W is the average body mass, which was 67.0 kg and 56.7 kg for male and female, respectively, according to the questionnaire data.

### 2.6 Non-carcinogenic risk

To assess the non-carcinogenic health risks, the hazard quotient (HQ) was calculated using Eq. 3:

$$\text{HQ}=\frac{\text{EDI}\times \text{SF}\times 0.001\;}{\;\text{RfD}}$$ (3)

where HQ is the hazard quotient; SF represents the safety factor, which was 10 in this study according to the National Science Foundation (NSF, 2003); RfD is the oral reference dose of the desired potentially toxic elements, with recommended values for Cd, As, Hg, and Cu of 0.001, 0.0003, 0.0001, and 0.5 mg/kg bw/day, respectively (USEPA, 2011; Mahmood and Malik, 2014). If the calculated HQ value is >1, the non-carcinogenic health risks of the exposure population cannot be ignored. Moreover, the total health risks caused by exposure to the multiple contaminants in earthworms were calculated using the hazard index (HI) based on Eq. 4:

$$\text{HI}={\text{HQ}}_{\text{Cd}}+{\text{HQ}}_{\text{As}}+{\text{HQ}}_{\text{Hg}}+{\text{HQ}}_{\text{Cu}}$$ (4)

### 2.7 Carcinogenic risks

The carcinogenic risk is described as the incremental probability of an individual developing cancer in their lifetime after exposure to a specific carcinogenic heavy metal (loid) (USEPA, 2009). Both As and Cd as are classified as human carcinogens (Zuo et al., 2020b).

The lifetime cancer risk (CR) for carcinogens was calculated by the cancer slope factor (CSF) using Eq. 5 (Islam et al., 2016):

CR=EDI×CSF×0.001 (5)

where CSF is oral cancer slope factor for carcinogens. According to the Integrated Risk Information System database, the recommended CSF for As and Cd is 1.5 and 6.1 (mg/kg/day)^−1^, respectively.

The total cancer risks caused by exposure to the multiple carcinogens were calculated using total lifetime cancer risks based on Eq. 6:

$$\text{TCR}={\text{CR}}_{\text{Cd}}+{\text{CR}}_{\text{As}}$$ (6)

In general, if the CR value is equal to or less than 10^−6^, carcinogenic effects are unlikely to occur over a lifetime (USEPA, 2009; USEPA, 2015; Gruszecka-Kosowska, 2018).

### 2.8 Probabilistic assessment through Monte Carlo simulation

Uncertainty may be present in deterministic risk estimations when using single-point input parameters during the risk assessment process. Thus, in our study a probabilistic assessment by Monte Carlo simulation (MCS) was explored to minimize the overall uncertainty of input variables (USEPA, 2001). MCS with 20,000 iterations was employed in R v.4.1.0 (R Core Team, Vienna, Austria) to handle uncertainties in the heavy metal (loid) contents, exposure frequency, and ingestion rate of earthworms. And the probabilistic distribution of the desired input variables was investigated. The values from these distributions were randomly selected and incorporated into the risk analysis. Through 20,000 iterations, the exposure models were simulated and the risks were calculated.

The development of an appropriate probability distribution of exposure variables for the studied potentially toxic elements will determine the reliability of the probabilistic risk results. In this study, we used face-to-face questionnaire data to develop the probability distribution of EF. The probability distribution of the IR of earthworms was based on the recommendations of the Chinese Pharmacopoeia. The concentration values of Cd, As, Hg, and Cu determined by ICP-MS were also simulated with the best probability distribution then inserted into the exposure assessment equations. The parameter values, optimum probability distributions of these parameters are presented in Supplementary Table S1.

### 2.9 Sensitivity analysis

The sensitivity analysis is an important part of the MCS, which allows the user to discern the extent of the variables with the greatest contribution to the total risks. During sensitivity analysis, one parameter is changed at a time, while all other variables remain constant (Gholizadeh et al., 2017). The Sobol sensitivity approach (SSA) used in this study is a variance-based approach to assess the contribution of each input variable and its interaction to the output model, with the results expressed as main effect and total effect, respectively (Fallahzadeh et al., 2017). The results of the sensitivity analysis are typically expressed as Sobol score, where higher scores indicate a greater impact of the parameters on health risks.

### 2.10 Statistical analysis

Figures were plotted using GraphPad software (version 5.0; San Diego, CA, United States) and the “ggplot2” package in R v.4.1.0 (R Core Team, Vienna, Austria). SPSS 19.0 (IBM Corporation, Armonk, NY, United States) and the “sensitivity” package in R v.4.1.0 (R Core Team, Vienna, Austria) were used to conduct the statistical analysis.

## 3 Results and discussion

### 3.1 Heavy metal (loid) content in earthworms determined via ICP-MS

**FIGURE 3:**
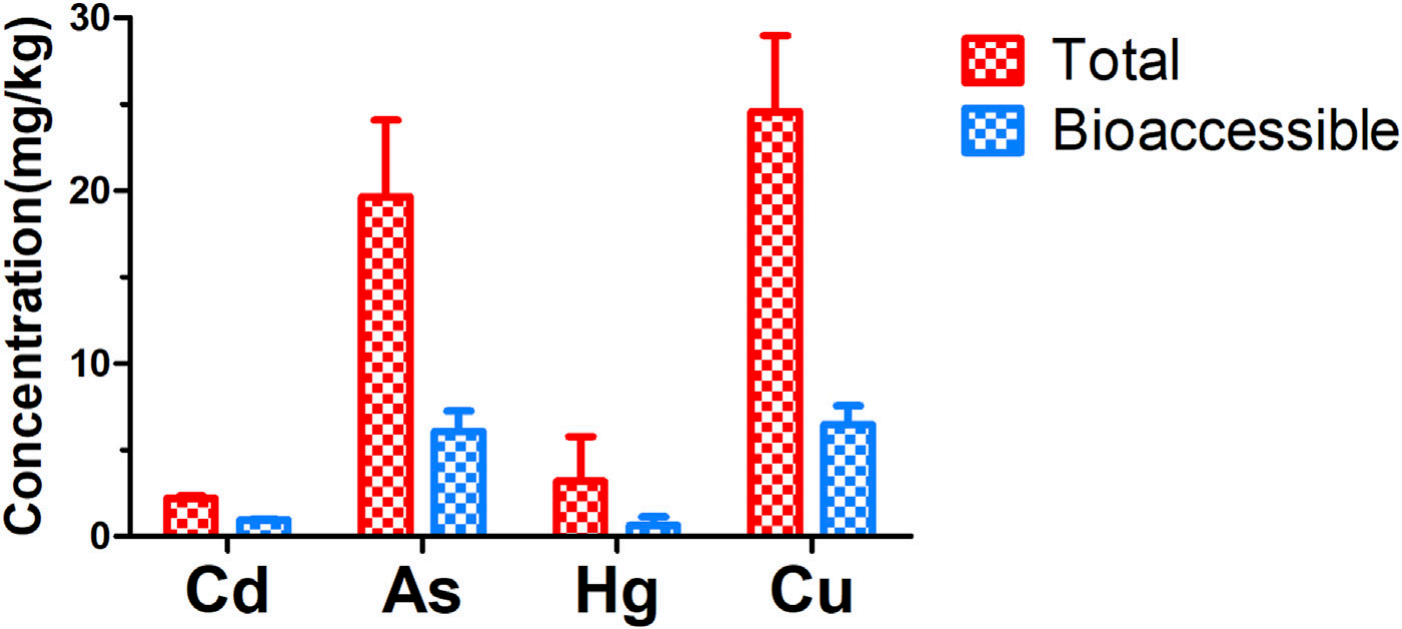
Mean concentrations of potentially toxic elements in earthworms from different origins.

As shown in Figure 3, the average concentrations of Cd, As, Hg, and Cu in 42 batches of earthworms were 2.22, 19.65, 3.18, and 24.58 mg/kg, respectively. According to the maximum permissible limit for leech, another type of traditional animal medicine, in the Chinese Pharmacopoeia, the average concentrations of As and Hg in earthworms were 3.9 and 3.2 times of the limit standard, respectively; therefore the risks of potentially toxic elements in earthworms may be of great concern.

The previous study reported that the arsenic concentrations of a total of 30 earthworm samples range between 0.4 and 53.6 mg/kg, which was comparable with our study (Li et al., 2022). Another study conducted on earthworms in Três Picos State Park in Rio de Janeiro, Brazil (0–20 cm soil depth) reported that the average Hg, concentrations in adult earthworm tissues ranged from 0.21 to 1.88 mg/kg (Buch et al., 2017), which was lower than the level detected in the present study. These heavy metal (loid) levels in earthworms were much higher than those in Chinese herbal medicines investigated in our previous study (Zuo et al., 2020b) and other published data on heavy metal (loid) contents in food or environment. For example, in a study in the Xinjiang Uygur Autonomous Region, Cd and As concentrations were determined to be 0.03–0.05 mg/kg and 0.10–0.45 mg/kg, respectively, in hops (*Humulus lupulus L.*) (Liu et al., 2019). The mean levels of As in rice in the Iranshahr market was 0.369 mg/kg, which was 0.02 times as the current study (Djahed et al., 2018). Additionally, the average Cd content in green tea from different geographical origins (India, China, and Japan) was only 0.015 mg/L, which was much lower than that in earthworms (Brzezicha-Cirocka et al., 2016). The much higher levels of potentially toxic elements in earthworms highlighted the necessity to study the bioaccessibility and scientifically investigate the associated health risks.

### 3.2 Bioaccessible concentration and bioaccessibility of potentially toxic elements

Compared to the total level, the bioaccessible concentration of potentially toxic elements in earthworms is a better index for representing the actual contents of potentially toxic elements readily absorbed by human body. The average bioaccessible potentially toxic elements contents in earthworms from different origins exhibited a broad range (Table 1). The mean bioaccessible concentration of potentially toxic elements in gastrointestinal phase of earthworms varied broadly as 0.44–1.73 mg/kg for Cd, 0.37–15.59 mg/kg for As, 0.13–7.13 mg/kg for Hg, and 3.42–12.79 mg/kg for Cu. The average bioaccessible values indicated that among the different origins, samples from Anhui, Guangxi, Guangdong and Guangdong were the highest accumulators of Cd, As, Hg, and Cu, respectively. The mean gastrointestinal bioaccessibility of Cd, As, Hg, and Cu in 42 batches of earthworms was in the following descending order: Cd (43.0%) >As (34.7%) >Cu (27.3%)> Hg (24.5%).

**TABLE 1:** Bioaccessible contents of potentially toxic elements in earthworms from different origins (Mean ± SD).

Origins	Batches	Contents of potentially toxic elements (mg/kg)			
		Cd	As	Hg	Cu
Shanghai	29	0.98 ± 0.36	5.35 ± 4.38	0.13 ± 0.20	6.22 ± 6.71
Shandong	3	0.79 ± 0.33	3.71 ± 2.02	0.18 ± 0.07	7.66 ± 3.14
Guangdong	3	1.05 ± 0.25	15.58 ± 20.30	7.13 ± 12.30	12.79 ± 16.95
Guangxi	2	0.44 ± 0.11	15.59 ± 20.97	0.29 ± 0.34	3.42 ± 0.99
Hainan	2	0.54 ± 0.06	0.37 ± 0.08	0.07 ± 0.04	4.26 ± 1.65
Jiangsu	1	0.98	4.21	0.04	5.01
Zhejiang	1	1.26	2.22	0.06	4.36
Anhui	1	1.73	4.27	0.07	5.89

### 3.3 Risk assessment

The outcomes of the risk assessment are prone to great uncertainties when the deterministic point estimation method is applied. To overcome these weaknesses, the probability distribution of crucial exposure variables in this study has been developed. Establishing an appropriate probability distribution of parameters is essential for obtaining reliable risk outputs. The probability distribution of IR and EF were based on questionnaire data to achieve a more realistic probabilistic model applicable to earthworms as well as to minimize potential errors. Additionally, given bioaccessibility of potentially toxic elements is more relevant to the actual potentially toxic elements levels exposed to the human body (Oomen et al., 2002; Poggio et al., 2009; Broadway et al., 2010; Liu et al., 2018), we focused on integrating the bioaccessibility of potentially toxic elements with the MSC technique.

The probabilistic outcomes based on bioaccessibility of potentially toxic elements are listed in Table 2. Among the potentially toxic elements, Cu had the highest EDI, with an average of 1.99 × 10^−2^ μg/kg/day and 1.83 × 10^−2^ μg/kg/day for male and female, respectively. However, Hg exhibited the lowest with an average of 6.65 × 10^−4^ μg/kg/day and 5.97 × 10^−4^ μg/kg/day for male and female, respectively. In general, for the high-exposure population (no less than 90th percentile), the estimated 90th percentile of the EDI exhibited the following order for the studied metal (loid)s: Cu > As > Cd > Hg. For high-exposure male, the 90th percentile of EDI for Cd, As, Hg, and Cu was 1.19 × 10^−2^, 5.34 × 10^−2^, 1.45 × 10^−3^, and 5.81 × 10^−2^ μg/kg/day, respectively, whereas that for high-exposure female was 1.04 × 10^−2^, 4.71 × 10^−2^, 1.32 × 10^−3^, and 5.16 × 10^−2^ μg/kg/day, respectively.

**TABLE 2:** Probabilistic estimation of EDI (μg/kg/d) and HQ of investigated potentially toxic elements.

			Minimum	P25	Mean	P50	P75	P90	P95	P99	Max
Cd	Male	EDI	2.46 × 10^−5^	3.50 × 10^−4^	4.13 × 10^−3^	1.32 × 10^−3^	4.94 × 10^−3^	1.19 × 10^−2^	1.76 × 10^−2^	3.22 × 10^−2^	7.98 × 10^−2^
		HQ	2.46 × 10^−4^	3.50 × 10^−3^	4.13 × 10^−2^	1.32 × 10^−2^	4.94 × 10^−2^	0.12	0.18	0.32	0.80
	Female	EDI	4.58 × 10^−5^	3.89 × 10^−4^	3.76 × 10^−3^	1.33 × 10^−3^	4.46 × 10^−3^	1.04 × 10^−2^	1.59 × 10^−2^	2.88 × 10^−2^	6.22 × 10^−2^
		HQ	4.58 × 10^−4^	3.89 × 10^−3^	3.76 × 10^−2^	1.33 × 10^−2^	4.46 × 10^−2^	0.10	0.16	0.29	0.62
As	Male	EDI	1.05 × 10^−5^	1.24 × 10^−3^	1.77 × 10^−2^	4.91 × 10^−3^	2.04 × 10^−2^	5.34 × 10^−2^	8.17 × 10^−2^	0.14	0.26
		HQ	3.50 × 10^−4^	4.13 × 10^−2^	0.59	0.16	0.68	1.78	2.72	4.73	8.51
	Female	EDI	1.24 × 10^−5^	1.36 × 10^−3^	1.63 × 10^−2^	5.03 × 10^−3^	1.85 × 10^−2^	4.71 × 10^−2^	7.29 × 10^−2^	0.13	0.26
		HQ	4.14 × 10^−4^	4.53 × 10^−2^	0.54	0.17	0.62	1.57	2.43	4.33	8.55
Hg	Male	EDI	2.33 × 10^−6^	3.27 × 10^−5^	6.65 × 10^−4^	1.31 × 10^−4^	4.97 × 10^−4^	1.45 × 10^−3^	2.78 × 10^−3^	9.68 × 10^−3^	2.51 × 10^−2^
		HQ	4.67 × 10^−5^	6.54 × 10^−4^	1.31 × 10^−2^	2.62 × 10^−3^	9.94 × 10^−3^	2.89 × 10^−2^	5.56 × 10^−2^	0.19	0.50
	Female	EDI	2.76 × 10^−5^	3.48 × 10^−5^	5.97 × 10^−4^	1.32 × 10^−4^	4.69 × 10^−4^	1.32 × 10^−3^	2.61 × 10^−3^	8.42 × 10^−3^	2.88 × 10^−2^
		HQ	5.52 × 10^−5^	6.95 × 10^−4^	1.19 × 10^−2^	2.65 × 10^−3^	9.38 × 10^−3^	2.64 × 10^−2^	5.22 × 10^−2^	0.17	0.58
Cu	Male	EDI	7.46 × 10^−5^	1.75 × 10^−3^	1.99 × 10^−2^	6.64 × 10^−3^	2.50 × 10^−2^	5.81 × 10^−2^	8.36 × 10^−2^	0.14	0.30
		HQ	1.86 × 10^−5^	4.37 × 10^−4^	4.97 × 10^−3^	1.66 × 10^−3^	6.24 × 10^−3^	1.45 × 10^−2^	2.09 × 10^−2^	3.54 × 10^−2^	7.45 × 10^−2^
	Female	EDI	1.41 × 10^−4^	1.96 × 10^−3^	1.83 × 10^−2^	6.66 × 10^−3^	2.22 × 10^−2^	5.16 × 10^−2^	7.63 × 10^−2^	0.13	0.33
		HQ	3.53 × 10^−5^	4.90 × 10^−4^	4.58 × 10^−3^	1.67 × 10^−3^	5.55 × 10^−3^	1.29 × 10^−2^	1.91 × 10^−2^	3.29 × 10^−2^	8.13 × 10^−2^

#### 3.3.1 Non-carcinogenic risk

**FIGURE 4:**
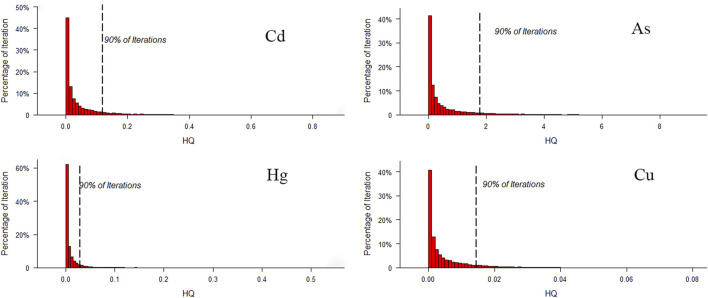
Simulation of HQ distribution of Cd, As, Hg, and Cu through 20,000 MCS iterations for male and female.

To evaluate the non-carcinogenic risks of the analyzed metal (loid)s, the HQ distributions based on bioaccessible concentration of potentially toxic elements were simulated by the MCS technique (Figure 4). The MCS technique is one of the most popular probabilistic models for health risk assessment because of its reliability and accuracy (Wang et al., 2007; Farzadkia et al., 2015; Miri et al., 2016a; Djahed et al., 2018; Fallahzadeh et al., 2018). A distribution of parameters instead of a fixed parameter value is applied in this risk model, enabling the stochastic behavior and probability distribution of the output to be investigated (Qu et al., 2016; Fallahzadeh et al., 2017; Ginsberg and Belleggia, 2017). The HQ results (Table 2) revealed that As exhibited the highest mean HQ, whereas the lowest was observed for Cu for both male and female. HQ values of As from the estimated 90th percentile to the maximum were greater than the threshold of 1 for both male and female, indicating an unacceptable non-carcinogenic health risks due to As exposure for high-exposure population. In short, for the generally exposed population (from P25 to P75), HQ values of Cd, As and Hg were <1, and the health risks were acceptable. However, for high-exposure population (from P90 to P99), the HQ values of As were >1 for both male and female, indicating an unacceptable non-carcinogenic health risks due to As exposure. From different origins, higher arsenic concentrations in samples from Guangdong and Guangxi contributed to higher HQ values.

**FIGURE 5:**
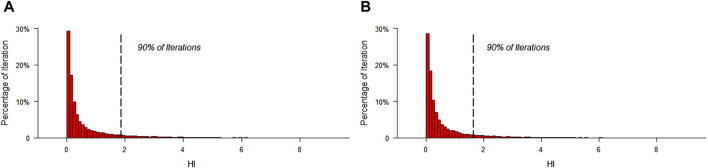
Simulation of HI distribution through 20,000 MCS iterations for male **(A)** and female **(B)**.

The mean HI values for all investigated metal (loid)s were below 1 for both male (0.65) and female (0.59). However, the 90th percentile of estimated HI values for the potentially toxic elements was 1.87 and 1.65 for male and female, respectively, which was not safe for humans (Figure 5). Moreover, the total non-cancer risks through exposure to the potentially toxic elements exceeded the acceptable level for 19.9% and 17.8% of male and female, respectively.

#### 3.3.2 Carcinogenic risk

**FIGURE 6:**
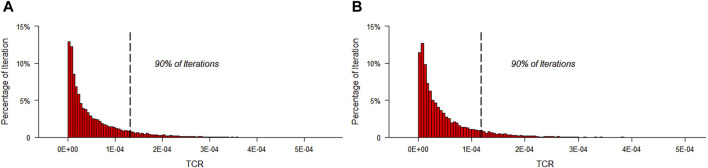
Simulation of total cancer risk probability distribution through 20,000 MCS iterations for male **(A)** and female **(B)**.

As and Cd are classified as carcinogens by the International Agency for Research on Cancer. Given the need to protect most consumers, it was assumed that all As species in earthworms were the most toxic inorganic forms. Based on bioaccessible concentration of potentially toxic elements, the outcomes of the MCS indicated that the mean CR values for As were 2.66 × 10^−5^ and 2.44 × 10^−5^ for male and female, respectively (Table 3). The mean CR values for Cd were 2.52 × 10^−5^ and 2.30 × 10^−5^ for male and female, respectively. Moreover, the total cancer risk (TCR) value through co-exposure to As and Cd were 5.18 × 10^−5^, 4,73 × 10^−5^ for male and female, respectively (Figure 6), which suggested that the carcinogenic risks may be of concern. A similar scenario was observed in a study in Iran, where the mean CR value for As was 2.37 × 10^−3^, indicating unacceptable carcinogenic risks in the investigated rice brands (Djahed et al., 2018). Our previous study on Chinese herbal medicines revealed that the CR values of As associated with several types of medicines, including plantain herb, argy wormwood leaf, Chinese angelica, morinda root, long tube ground ivy herb, and dyer woad leaf, were higher than the permissible level for the high-exposure population (Zuo et al., 2020b).

**TABLE 3:** CR values of Cd and As.

		Minimum	P25	Mean	P50	P75	P90	P95	P99	Max
Cd	Male	1.50 × 10^−7^	2.14 × 10^−6^	2.52 × 10^−5^	8.07 × 10^−6^	3.01 × 10^−5^	7.23 × 10^−5^	1.07 × 10^−4^	1.96 × 10^−4^	4.87 × 10^−4^
	Female	2.80 × 10^−7^	2.37 × 10^−6^	2.30 × 10^−5^	8.10 × 10^−6^	2.72 × 10^−5^	6.35 × 10^−5^	9.07 × 10^−4^	1.76 × 10^−4^	3.79 × 10^−4^
As	Male	1.58 × 10^−8^	1.86 × 10^−6^	2.66 × 10^−5^	7.37 × 10^−6^	3.06 × 10^−5^	8.01 × 10^−5^	1.23 × 10^−4^	2.13 × 10^−4^	3.83 × 10^−4^
	Female	1.86 × 10^−8^	2.04 × 10^−6^	2.44 × 10^−5^	7.55 × 10^−6^	2.77 × 10^−5^	7.07 × 10^−5^	1.09 × 10^−4^	1.95 × 10^−4^	3.85 × 10^−4^

### 3.4 Sensitivity analysis

**FIGURE 7:**
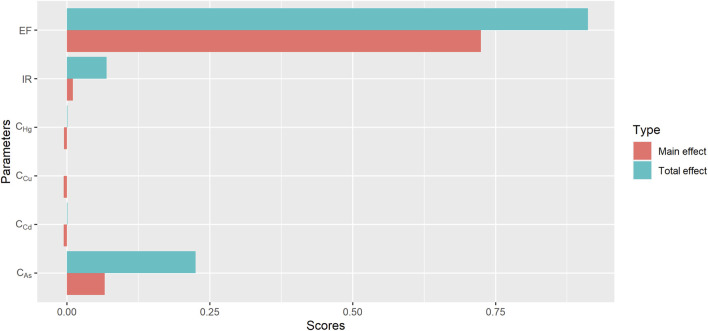
Sensitivity analyses of exposure frequency (EF), daily ingestion rate (IR) and concentrations of potentially toxic elements for HI.

Sensitivity analysis offers a quantitative ranking of the input parameters based on their contributions to the variability and uncertainty of the model output (USEPA, 2001). Figure 7 shows the sensitivity analysis results of the most important parameters influencing the estimated risk outputs. We determined that the EF of individuals to earthworms was the most important variable affecting the results, followed by the bioaccessible concentrations of As. Another study on the human health risk of chromium in mangrove sediments and toxic elements in groundwater sources in southwestern Nigeria found that contaminant concentration and exposure frequency were the two most sensitive exposure parameters affecting the estimation of output results as well (Emenike et al., 2019). Therefore, refining the exposure frequency of earthworms and decreasing the concentration of the studied potentially toxic elements in earthworms are identified as the two most beneficial suggested environmental management methods.

### 3.5 Remediation strategies and prospective research to improve ecological and public health

Given potential risks of earthworm ingestion cannot be neglected for high-exposure populations, environmental management policies should be implemented to minimize risks with the main goal of improving the population health through the scientific use of earthworms. Firstly, emission reduction of potentially toxic elements and the remediation of areas contaminated with potentially toxic elements should be considered. Among the assessed potentially toxic elements, As should be preferentially managed owing to its relatively high health risks. Secondly, the sensitivity analysis results revealed that the exposure frequency and bioaccessible concentrations of As in earthworms were the most influential variables in the probabilistic risk assessment, therefore the refinement of earthworm exposure frequency and further monitoring of heavy metal (loid) contents are highly recommended. Thirdly, in the interest of ecological and public health improvement, enhanced awareness of environmental protection and remediation of areas contaminated with potentially toxic elements are recommended. Protection of ecological environments where TCM components are sourced and humans live is suggested. Furthermore, remediation involving physical, chemical, and biological remediation strategies are encouraged to remove or control potentially toxic elements in contaminated sites (Sodhi et al., 2022). Physical and chemical approaches include adsorption, membrane filtration, electrodialysis, chemical precipitation, and photocatalysis (Dakal et al., 2016; Jacob et al., 2018). However, these conventional methods are constrained by high cost, processing problems, and the generation of toxic sludge. Therefore, biological remediation strategies involving the use of bacteria, fungi, plants, and diatoms for the removal of metal ions from the environment should be further explored considering that they are more socially acceptable, economical, and environmentally friendly than many conventional methods (Abatenh et al., 2017; Chandrangsu et al., 2017). Moreover, since variations in the nutritional status, dietary structure, and metabolism of different exposed individuals may lead to uncertainties of the risk assessment outcomes, it is encouraged to explore the combination of MCS technique and population-based biokinetic models to better evaluate the dynamic health risks of environmental contaminants and protect public health in future.

## 4 Conclusion

In the present study, we developed a novel and refined probabilistic risk assessment strategy for evaluating the risk of potentially toxic elements exposure from consuming earthworms based on bioaccessibility and the MCS technique. Moreover, to obtain a more realistic scenario, the EF and Ed to earthworms were obtained from questionnaire data, and the safety factor was applied to construct an assessment model that was applicable to earthworms. The mean gastrointestinal bioaccessibility of potentially toxic elements was in the order: Cd > As > Cu > Hg. The non-carcinogenic risk assessment findings indicated that the health risks of earthworm ingestion are acceptable for populations with average exposure. However, the 90th percentile HI values suggested that more attention should be paid to protecting populations with high exposure. In addition, the TCR value from co-exposure to As and Cd suggested that the carcinogenic risks may be of concern for populations with average exposure. Sensitivity analyses revealed that the EF and bioaccessible concentration of As were dominant contributors to the total risk variance. Overall, this study demonstrated the applicability of stochastic exposure assessment methods based on PBET-extracted bioaccessibility in precisely and scientifically determining the health risks of potentially toxic elements present in earthworms. This study provides novel perspectives for minimizing risks and improving the safety of TCM, facilitating the scientific use of TCM to treat complex diseases in clinical settings. For further research, it is suggested to develop limit standard of potentially toxic elements based on bioaccessibility but not total contents in TCM with the consideration of different exposure population.

## Data Availability

The original contributions presented in the study are included in the article/Supplementary Material, further inquiries can be directed to the corresponding author.

## Abbreviations

TCM, traditional Chinese medicine; MCS, Monte Carlo simulation; EDI, estimated daily intake; ICP-MS, inductively coupled plasma mass spectrometry; HQ, hazard quotient; HI, hazard index; CR, lifetime cancer risk; TCR, total cancer risk; CSF, cancer slope factor.
