# Supplementary material for: A novel bioaccessibility-based probabilistic risks assessment of potentially toxic elements (PTEs) in earthworm
Source: Front Pharmacol. 2024 Jul 4;15:1398394. doi: 10.3389/fphar.2024.1398394 (PMC11254821; doi:10.3389/fphar.2024.1398394)
Supplement: Supplementary file 1 [file Table1.docx]

Table S1. The optimum probability distribution of the parameters simulated in Monte Carlo simulation technique

| Parameter | Ed | BA | AT | W | EF | IR | C (mg/kg) | | | |
| --- | --- | --- | --- | --- | --- | --- | --- | --- | --- | --- |
|  |  |  |  |  |  |  | Cd | As | Hg | Cu |
| Values or type of distribution | 20 (year) | Detected values (%) | 25550  (day) | 67.0 (kg) for male and 56.7 (kg) for  female, respectively | Beta binomial distribution | Uniform distribution | Lognormal distribution | Simple random sampling | Simple random sampling | Weibull distribution |
